# Development of a human machine interface for robotically assisted surgery optimized for laparoscopic workflows

**DOI:** 10.1007/s11548-024-03239-3

**Published:** 2024-08-10

**Authors:** Luca Wegener, Dirk Wilhelm, Maximilian Berlet, Jonas Fuchtmann

**Affiliations:** 1 Klinikum rechts der Isar, Research Group MITI, TUM School of Medicine and Health, Technical University of Munich, Munich, Germany; 2 Klinikum rechts der Isar, Department of Surgery, TUM School of Medicine and Health, Technical University of Munich, Munich, Germany; 3 Munich Institute of Robotics and Machine Intelligence, Technical University of Munich, Munich, Germany

**Keywords:** Human machine interface, Input device, Robotic-assisted surgery, Surgical workflow

## Abstract

****Introduction**:**

In robotic-assisted surgery (RAS), the input device is the primary site for the flow of information between the user and the robot. Most RAS systems remove the surgeon’s console from the sterile surgical site. Beneficial for performing lengthy procedures with complex systems, this ultimately lacks the flexibility that comes with the surgeon being able to remain at the sterile site.

****Methods**:**

A prototype of an input device for RAS is constructed. The focus lies on intuitive control for surgeons and a seamless integration into the surgical workflow within the sterile environment. The kinematic design is translated from the kinematics of laparoscopic surgery. The input device uses three degrees of freedom from a flexible instrument as input. The prototype’s performance is compared to that of a commercially available device in an evaluation. Metrics are used to evaluate the surgeons’ performance with the respective input device in a virtual environment implemented for the evaluation.

****Results**:**

The evaluation of the two input devices shows statistically significant differences in the performance metrics. With the proposed prototype, the surgeons perform the tasks faster, more precisely, and with fewer errors.

****Conclusion**:**

The prototype is an efficient and intuitive input device for surgeons with laparoscopic experience. The placement in the sterile working area allows for seamless integration into the surgical workflow and can potentially enable new robotic approaches.

## Introduction

Today, minimally invasive surgery (MIS) is regarded as an integral part of medicine. Compared to open surgery, it results in less pain, faster rehabilitation, creates vastly better cosmetic results, and has a lower rate of complications [1].

With laparoscopy being a standard procedure, the urge to improve the clinical outcome of surgeries calls for a further reduction of trauma during invasive surgical procedures [2]. Resulting developments have been transluminal and single port surgery [3, 4]. Medical devices developed for these procedures were innovative and pushed new possibilities but failed to prevail in the market. Most devices are complicated to use, prolong the procedure, and present no competition to laparoscopy [5]. Recently developed systems have aimed to solve these problems using robotic approaches. With STRAS, the motorization of the degrees of freedom (DoF) aims at better control of a platform initially intended for pure mechanical control [6, 7]. The PLAFOKON platform implements a patient and procedure individual snake-like surgical manipulator called SPOT that is produced by additive manufacturing [8].

While some robotic-assisted surgery (RAS) systems use specifically designed kinematics for the input human–machine interface (HMI), others use HMI that are commercially available [9]. The Da Vinci Surgical System (Intuitive Surgical, Sunnyvale, California, USA) utilizes specifically designed serial kinematics for the input device on the surgeon’s console [10]. The STRAS initially utilized an omega.7 (Force Dimension, Nyon, Switzerland) [6]. As accurate models without singularities for Cartesian control were hard to create, a custom kinematic input interface was built [7]. Systems in research often include devices from Force Dimension, the Phantom Omni, and Phantom Premium (3D Systems GmbH, Moerfelden-Walldorf, Germany). For the neuroArm, two PHANTOM Premium 1.5 were modified according to the requirements for stereotaxy and microsurgery in neurosurgery [11]. Phantom Omni is incorporated into the RAVEN platform. [12] Force Dimension manufactures a broad portfolio of devices with varying input DoFs, but all share the characteristic parallel kinematics. Their flagship model, the sigma.7, was designed in cooperation with the DLR for the MiroSurge system [13].

All of these systems follow the concept of tele-manipulation systems, where the surgeon’s console is removed from the sterile surgical site [1]. This concept of a separate surgeon’s console can be beneficial for complex systems when performing lengthy procedures. However, ultimately, they lack the flexibility that comes with the surgeon being able to remain at the sterile site while controlling a robotic device [9]. Especially with smaller robotic systems, a separate console that is removed from the surgical procedure could inhibit the surgical workflow. Our approach, therefore, is to develop an HMI for the surgeon to use from the sterile field.

## Methods

### Individual approach

To enhance the accessibility of robotic systems and truly make use of their benefits, it is necessary to incorporate RAS into the surgical workflow. The main focus hereby must be a perfectly intuitive control interface for the surgeon. A simple-to-use HMI that gives control of the degrees of freedom of a robotic surgical device is essential for future robotic interventions. Such an HMI needs seamless integration into the surgical workflow, as we aim for a system that can be used by the surgeon at the surgical site. A compact and easy-to-use system requires less setup time and would be a more cost-effective solution than the current tele-manipulation devices. In this paper, a novel approach of an HMI for RAS is presented that is developed around the following principles:

- Seamless integration into surgical workflow
- Intuitive control by surgeons
- Using an endoscopic instrument as input
- Generic usage for different surgical robotic platforms with thee DoF is striven

The prototype is developed around the idea of utilizing an endoscopic instrument as the means of input. Flexible endoscopic instruments have decoupled DoF of the handle and the tip of the instrument. The use of such instruments in RAS has been demonstrated successfully for abdominal surgery in the SPOT system [8]. With these decoupled mechanics, the free DoF of the handle can be measured by sensors inside the prototype HMI and used to control as much as three DoF of a robotic surgical device. Applications for such an input device could include the guidance of a laparoscopic camera with a robotic system (e.g., SOLOASSIST II, AKTORmed GmbH, Neutraubling, Germany) or the control of robotic instruments with three DoF (e.g., endoscopic instruments of STRAS).

**Fig. 1:**
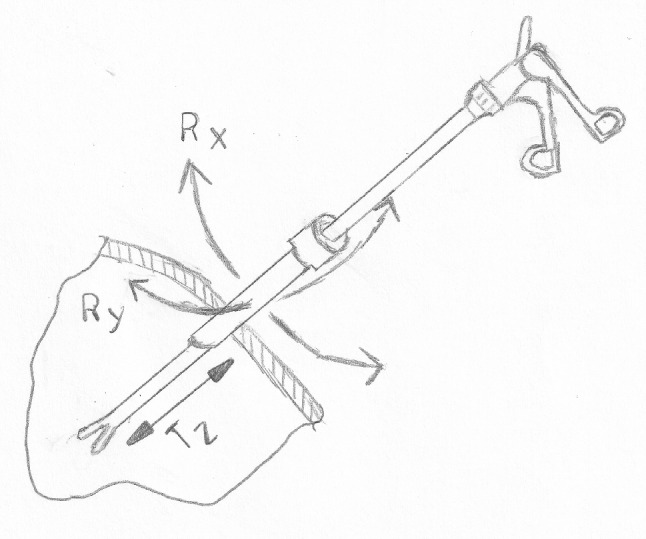
Kinematics of laparoscopic surgery: $R_x$ pivotal rotation around the x-axis, $R_y$ pivotal rotation around the y-axis, $R_z$ rotation around the z-axis, $T_z$ translation along the z-axis

**Fig. 2:**
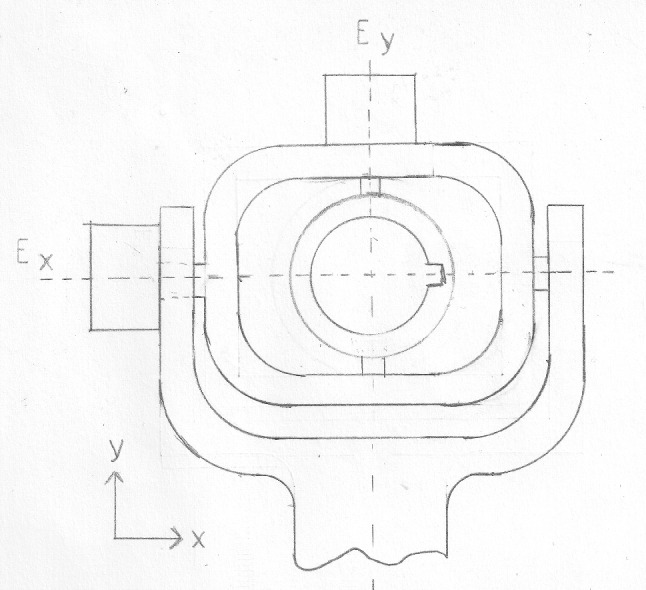
Draft of the concept for the kinematic design of the prototype ($E_x$: x-axis of prototype, $E_y$: y-axis of prototype, z-axis orthogonal to the plane)

The tenet of the concept is to utilize the kinematics that surgeons are accustomed to. During laparoscopy, the instrument is inserted through a trocar, which creates a pivot point. As illustrated in Fig. 1 an instrument during surgery has four DoF: rotation along the x-, y- and z-axis ($R_x$, $R_y$, $R_z$) as well as the translation along the z-axis ($T_z$). Using these degrees of freedom as the input method for the prototype would make the control for the surgeon intuitive and easy to use. The surgeon can now think of the virtual tooltip or a robotic end-effector as if it were a laparoscopic instrument. The hardware development focuses on translating these kinematics of laparoscopy to an HMI that can be used for RAS. The DoF that are used for the prototype are the rotation around the x-axis ($R_x$) and the y-axis ($R_y$), as well as the translation along the z-axis ($T_z$). A draft of the kinematic design is shown in Fig. 2. The rotation around the instrument $R_z$ is not utilized in the concept since this DoF is not decoupled for a flexible instrument. The range of motion of this prototype was identified through an iterative process with senior surgeons, resulting in a range of motion around the x-axis and y-axis of $\pm 30^{\circ }$ and along the z-axis of $\pm 100 mm$.

For seamless integration into the surgical workflow, positioning the prototype in the sterile area is assumed to be essential. A sterility concept is developed that influences the design process. The instrument is easily attachable and detachable from the HMI in case of a medical complication.

Integration of the prototype requires a flexible software interface. A suitable framework for the implementation of the required features is the robot operating system (ROS). ROS 2 in combination with C++ is chosen for the implementation as it has several key features over the first version of the framework and performance advantages. The encoders are connected via microcontrollers that run micro-ROS and integrate into the ROS ecosystem.

### Construction of the prototype

For construction, machining and additive manufacturing are used. The frame is made of machined aluminum *EN AW-5083* which provides rigidity while staying compact. Any parts with complex geometries and delicate design features are produced using additive manufacturing. These parts were manufactured with either the *Form 3B* or the *Form 2* (*Formlabs GmbH*, Somerville, USA). Depending on the mechanical requirements the material used are *White V4*, *Clear V4*, *Grey Pro V1*, *Tough V5* and *Durable V2*.

The design of the hardware focusses around the four central ideas: Integration into the surgical workflow, intuitive control, using an endoscopic instrument as input and a generic usage for different robotic systems. It incorporates two rotational encoders for encoding the movement around the x-axis and one translational encoder for the z-axis. It is designed with the bottom-up design principle around the instrument and encoders.

The instrument is connected to the prototype via a simple, robust, and reliable connection interface. It is vital that if any complication occurs during surgery, the instrument can be detached from the main frame quickly and intuitively. An adapter mounts onto the instrument by hand via friction and connects to the connection interface. The instrument with the adapter can be pushed into the interface and is locked into place by a locking bolt. By lifting the bolt, the instrument can be fully detached from the prototype. The adapter is additively manufactured and thought to be a single-use product. For the usage with different endoscopic instruments, the inner geometry of the adapter can be modified without the need to adapt the prototype or connection interface itself.

The compact and modular magnetic linear encoder (RLM Miniature Incremental Magnetic Encoder, *RLS Merilna tehnika d. o. o., Komenda, Slovenia*) is used to encode the movement along the z-axis. A magnetic encoder is robust in regards to contamination. It uses a magnetic strip that is embedded into the sliding frame. The sliding frame is housed by the main structural frame of the z-assembly, which is machined from aluminum. It connects to the y-axis on the top and bottom.

**Fig. 3:**
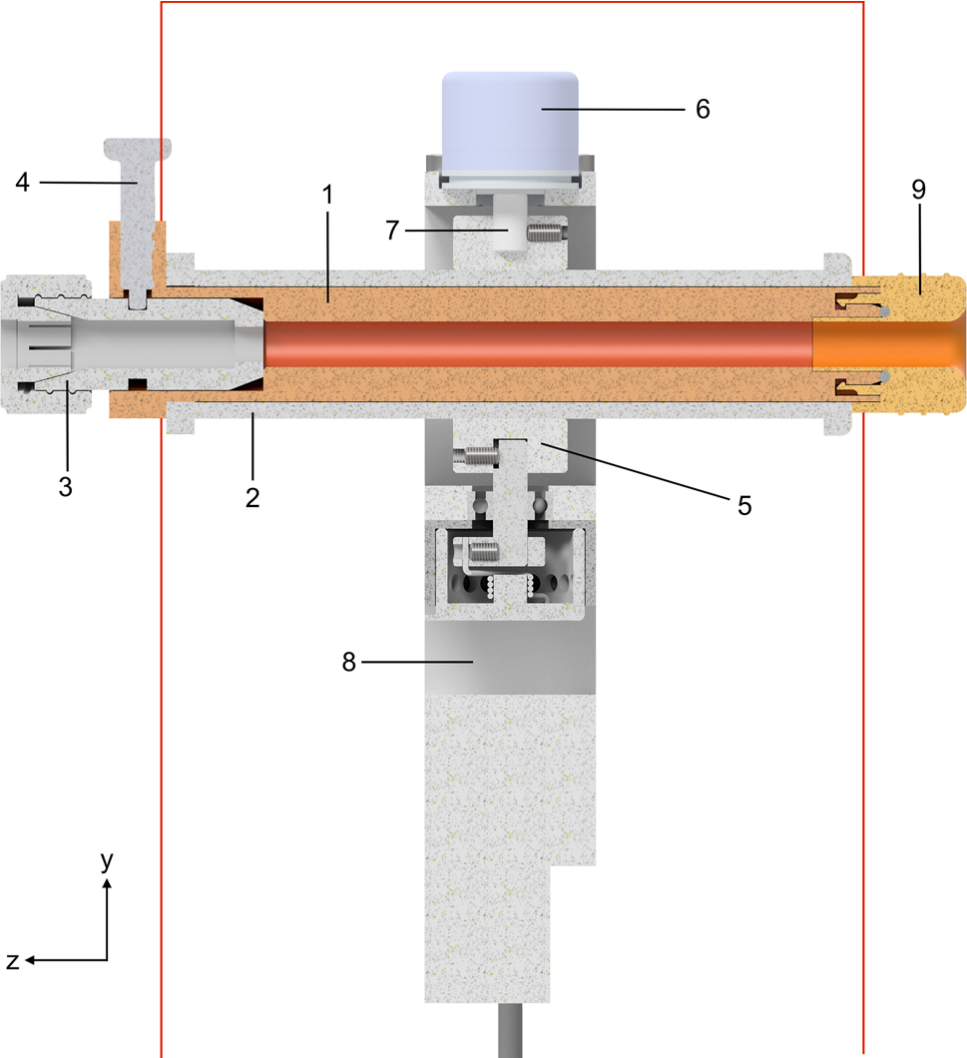
Cross section of the prototype (red line illustrates the sterile plastic cover): (1) sterile insert (dark orange); (2) sliding frame with magnetic strip; (3) connection adapter for the instrument; (4) locking bolt; (5) structural frame of the z-assembly housing z-encoder; (6) rotational encoder y-axis; (7) connection to the y-axis; (8) main frame of the prototype; (9) end cap (light orange)

**Fig. 4:**
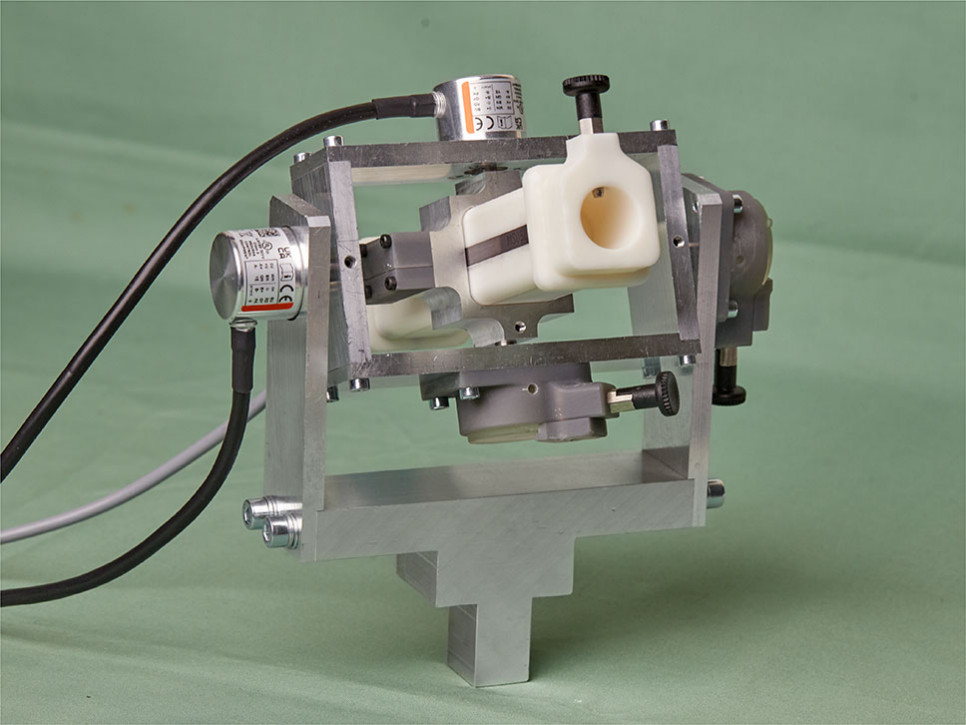
Complete assembly of the prototype

**Fig. 5:**
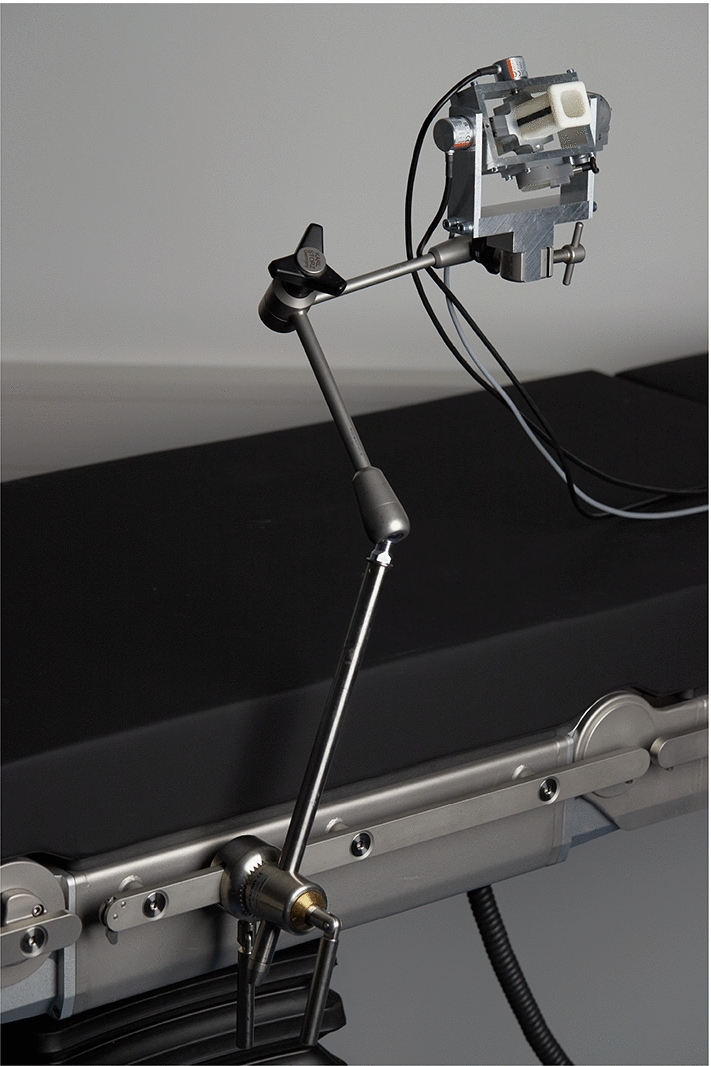
Prototype mounted to operating table

To encode the x-axis and y-axis, enclosed optical encoders (*2400 series*, *Kuebler Group, Fritz Kuebler GmbH, Villingen-Schwenningen, Germany*) are chosen. The x and y-assembly each consist of a rectangular frame that houses the respective encoder and are built around each other. Fixed-floating bearing configurations with sealed roller bearings are chosen for both rotational axes. In Fig. 3, the cross-section of the prototype shows the z-assembly connected to the y-assembly through the shafts. A torsional spring provides a retention force for both axes. The zero point of the retention force can be set, a locking bolt on each axis can completely deactivate it. The x, y, and z-assembly are nested and mounted together to form the complete prototype in Fig. 4. The prototype can be attached to the operating table with a holding arm via the standard rail mounts (see Fig. 5). This makes a flexible positioning in the sterile area possible.

The positioning of the prototype in the sterile area is thought to be key for the seamless integration into the surgical workflow, but mandates a concept for maintaining the sterility. The instrument has to maintain its sterility when inserted in the prototype, and the prototype cannot contaminate the sterile area. A design using a sterile plastic cover is chosen. As illustrated in Fig. 3 by the red line, the cover encloses the prototype. To ensure that the instrument maintains sterility, it is only in contact with single-use sterile parts. For assembly, a new sealed sterile insert is inserted through the prototype at the beginning of each surgery. Once it is inserted, the seal is broken, and the end cap is clipped onto the sterile insert. Both the sterile insert and the end cap are welded onto the plastic cover and completely encapsulate the prototype while allowing a sterile working channel through the prototype for the instrument. The instrument can be detached and reattached through the connection interface while maintaining sterility.

The encoders are each connected to an Arduino Due that runs micro-ROS. Each Arduino runs a ROS node and submits the current value of the encoder to their respective topic. A main computational node subscribes to the topics, computes the value, and provides an interface through which the values can be transmitted to a robotic actuator. With ROS, the interface is flexible and can be adapted to different systems.

### Evaluation

**Fig. 6:**
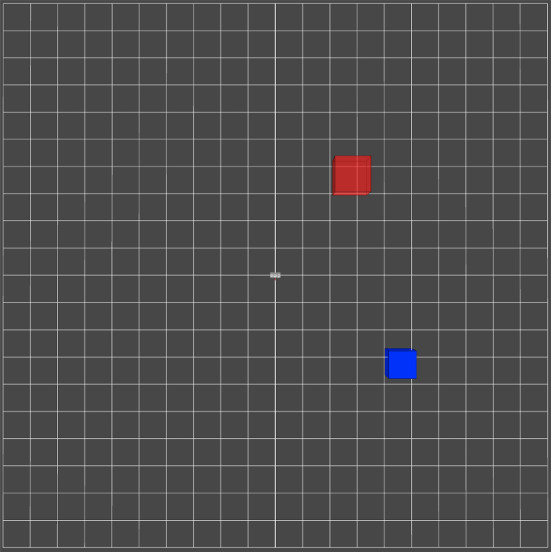
Task **Target**: The input marker (blue) needs to navigate to the target marker(red)

**Fig. 7:**
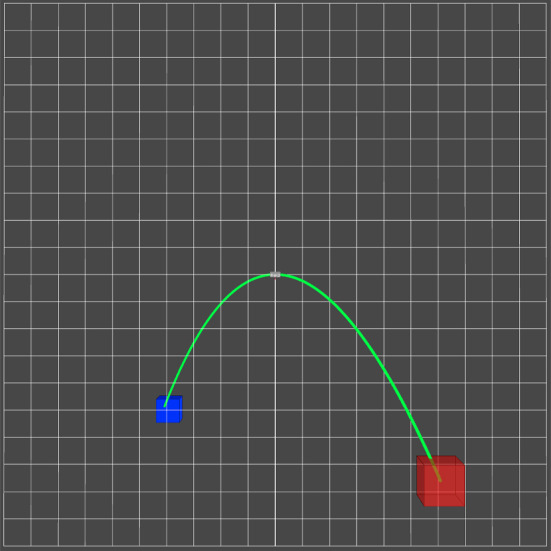
Task **Trace Line**: The input marker (blue) needs has to accurately move along the line (green) toward the stop marker (red)

In a first experimental study, it is evaluated how the prototype compares to a commercially available HMI (*SpaceMouse, 3Dconnexion GmbH*, Munich, Germany) during the usage by surgeons (n=12) to gain a rough understanding of our prototype. The evaluation of the prototype solely focuses on the input devices themselves, and thus, it is evaluated in a simplified virtual environment without the use of a robotic actuator. This ensures that the robotic actuator has no influence on the results. Evaluation metrics proposed in the literature are used to evaluate the performance. The virtual environment is created with the ROS package foxglove. In this environment, two tasks are designed to measure the surgeons’ performance. The surgeon controls an input marker with the input device. In the first task **Target** (see Fig. 6), the surgeon has to consecutively overlap target markers in three-dimensional space with the input marker in as little time as possible. The second task **Trace Line** is designed to evaluate the precision. The surgeon has to precisely follow the line from start to finish in the three-dimensional space. For each time the marker leaves the line, an error is counted. Each surgeon repeats the tasks three times (Fig. 7).

Over time, different metrics have been proposed, refined, developed, and tested for different tasks in a number of studies [14]. These metrics can be divided into two categories [15]:

- *Efficiency metrics* These quantitative metrics are measurable physical parameters precisely defined and supported by a robust theoretical background [14]. They are calculated automatically in physical or virtual reality simulators or by tracking the movement of a surgical tool during surgery [15].
- *Quality metrics* Depending on the definition and the procedure of the task, these metrics describe the quality of the execution of the task [14]. A common quality metric is assessing the errors during the execution of a task regarding their number, frequency, and degree of importance [15, 16].

Metrics are selected depending on their relevance in literature and if a construct validity has been proven in studies. As the prototype is designed with three degrees of freedom corresponding to translational movement, only metrics with the tool center point (TCP) position as variables are selected.Data of the evaluation is recorded with *rosbag2*, and the metrics are computed with MATLAB ((R2023a; *The MathWorks, Inc.*, Natick, USA). First, the selected efficiency metrics are presented.

During a task, the position *X* of the input marker that simulates the TCP is described by a finite number N of discrete points:

1 $$\begin{aligned} X = \left[ \begin{matrix} x[n] \\ y[n] \\ z[n] \end{matrix} \right] _{n=0}^{N} \end{aligned}$$

The tasks start at *t*(0) and end at *t*(*N*), where *t* is the timestamp of the ROS real-time clock.

1. *Time (T)*: The time from the task’s start to its completion, measured in seconds. [14, 16–20]
  2 $$\begin{aligned} T = t[N] - t[0] \end{aligned}$$
2. *Path length (PL)*: The total movement of the tip of the instrument or TCP during the task’s duration. [14, 18–20]

3 $$\begin{aligned} PL{}&= \sum _{n=1}^{N-1} \left|\Delta X \right|_{2} = \sum _{n=1}^{N-1} \left|\left[ \begin{matrix} x[n] \\ y[n] \\ z[n] \end{matrix} \right] -\left[ \begin{matrix} x[n+1] \\ y[n+1] \\ z[n+1] \end{matrix} \right] \right|_{2} \nonumber \\&= \sum _{n=1}^{N-1} \sqrt{ \bigl (x[n]-x[n+1]\bigr )^2 + \bigl (y[n]-y[n+1]\bigr )^2 + \bigl (z[n]-z[n+1]\bigr )^2 } \end{aligned}$$

**Fig. 8:**
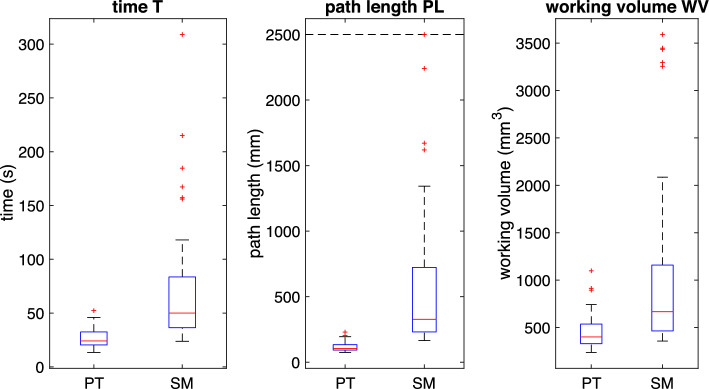
Calculated metrics of the task **Target**, visualized as a boxplot (PT: Prototype, SM: SpaceMouse; n = 12)

**Fig. 9:**
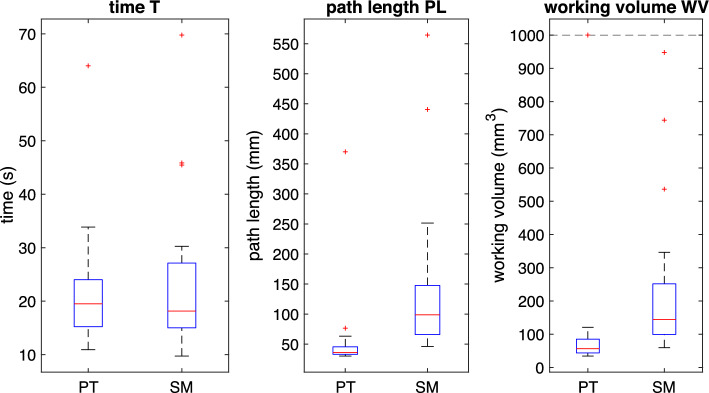
Calculated metrics of the task **TraceLine**, visualized as a boxplot (PT: Prototype, SM: SpaceMouse; n = 12)

**Fig. 10:**
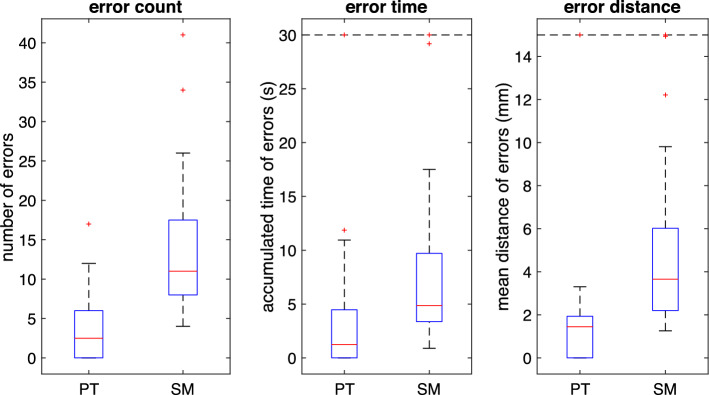
Error data of the task **Trace Line**, visualized as a boxplot (PT: Prototype, SM: SpaceMouse; n = 12)

3. *Working Volume (WV)* Forming a convex hull around the trajectory of the TCP, the volume of this hull represents the working volume. Despinoy et al. use the MATLAB (The MathWorks Inc, Natick, USA) function convhull() to calculate this metric. [20]
  4 $$\begin{aligned} WV = \texttt {convhull}\left( X \right) = \texttt {convhull}\left( \left[ \begin{matrix} x[n] \\ y[n] \\ z[n] \end{matrix} \right] _{n=0}^{N} \right) \end{aligned}$$

In addition to these efficiency metrics, the following error metrics are computed for the task **Trace Line**. An error occurs when the input marker’s volume no longer touches the line.

1. *Number of errors* The number of errors counts how often a surgeon has left the line with the volume of the input marker no longer touching the line.
2. *Accumulated time of the error* The time in seconds counts how long the input marker has left the line for each error. The sum of the error durations from one evaluation instance represents the accumulated time of the error.
3. *Mean error distance* When an error occurs, the distance the marker diverts from the line is averaged throughout each error.

A statistical analysis of the computed results is conducted. The Shapiro–Wilk test shows at a significance level of $\alpha =1\%$ that a normal distribution at the selected significance level can not describe most data groups. As there are only two data groups, there is no need for a Friedman’s test, and the Wilcoxon rank sum test is directly applied without a p-value adjustment. The significance level is selected as $\alpha =1\%$.

## Results

The results for the task **Target** show a considerable difference in the performance metrics when the surgeons use the prototype compared to the SpaceMouse. It can be seen in Fig. 8 that the surgeons need less time to complete the task with the SpaceMouse. In addition, they can complete the task using a shorter path length and a smaller working volume. The difference in median of all three metrics is statistically significant (see Table 1).

For the task **TraceLine**, the boxplot in Fig. 9 shows that the surgeons need a similar amount of time to complete the task with both input devices. The statistical analysis (Table 1) reveals no statistically significant difference in the median for the time T. In contrast, the differences in the path length and the working volume the surgeons need show a statistically significant difference.

Figure 10 shows that the surgeons make significantly more errors using the SpaceMouse than when they use the prototype. In addition, the errors that occur are longer, and the distance that the surgeon diverts from the path is significantly longer. The difference in the median of these results is statistically significant (Table 2).

## Discussion

The evaluation results show that the surgeons can complete the first task faster with the prototype than with the SpaceMouse. There is no such difference in the second task, TraceLine, and the surgeons need a comparable amount of time to complete the task. The second task focuses much more on a combined evaluation of precision and time, as there were error points given, and fast but sloppy performance is not incentivized. When combining these two factors, the conclusion can be drawn that time alone cannot be used to determine how the input device performs. However, it can be considered when looking at the performance metrics.

The significantly shorter path length in both tasks indicates a higher precision and the prototype being more intuitive to use for surgeons. Shorter, more direct paths are taken by the surgeons, leading to a faster completion of the procedure. Furthermore, there is a significant difference in working volume, as the surgeons need a smaller volume to complete the tasks with the prototype. This reinforces the hypothesis of more direct and efficient paths when using the prototype. It must be highlighted that while the surgeons need a similar amount of time to follow the line, they have more precise movements with the prototype.

The error metrics underpin these results. The surgeons make fewer errors with the prototype. This means fewer uncontrolled movements make them divert from the path. One can come to the same conclusion when looking at the mean error distance. It is much lower when using the prototype than with the SpaceMouse. Furthermore, the time it takes the surgeons to correct the mistake and maneuver the input marker back onto the curve is much shorter for the prototype.

The hypothesis can be made that the more intuitive kinematics of this HMI lead to a more familiar and precise interface for surgeons.

This first prototype demonstrates how an easy-to-use HMI could be integrated into the surgical workflow. Precisely controlling robotic devices from the surgical site while maintaining sterility is a key benefit of this approach. The most commonly used RAS, the daVinci, uses a separate surgeon’s console outside the sterile working area. When performing lengthy procedures for complex surgeries, such a system design can be beneficial. However, such systems are not widely used for shorter and less complex surgeries at the moment. For these procedures, smaller robotic systems (e.g., camera robots) could potentially integrate better into the workflow. Especially for these smaller robotic systems, a separate console removed from the surgical procedure could inhibit the surgical workflow. While the input kinematics of e.g., the daVinci have been developed over decades and provide a high accuracy and reliability, the developed experimental prototype demonstrates a new flexible and intuitive HMI that could be beneficial in future robotic applications in surgery as it provides a better integration into the surgical workflow. In a first experiment, the derivation of the DoF from laparoscopy has proven beneficial and enabled an intuitive control for surgeons. Translating the kinematics of laparoscopy to robotic surgery could provide a benefit for easier control. In future studies, the prototype has to be evaluated regarding surgical tasks, e.g., suturing, and compared to certified input devices currently applied in RAS. Furthermore, it is aimed to conduct pre-clinical and clinical studies to fully understand the benefits and limitations of the prototype in real-world clinical scenarios.

The prototype integrates a barrier between the electro-mechanical components and the sterile surroundings to maintain sterility at the sterile surgical site. While providing a theoretical maintenance of sterility, this concept has to be evaluated further regarding reliability. For easy integration, the sterile assembly can be mounted without using additional tools directly to the rails of the operating table. The prototype’s connection interface and adapter proved reliable during the study and can be adapted to the geometry of different instruments. It provides a flexible and efficient way of connecting an instrument to the prototype.

## Conclusion

In the scope of this work, a prototype of a novel input device for RAS is designed and constructed. The design focuses on intuitive control by the surgeon and seamless integration into the laparoscopic workflow. The prototype utilizes the input of an endoscopic instrument. A possible use case is in combination with the SPOT system. The direct positioning at the sterile surgical site ensures seamless integration into the surgical workflow. The prototype is evaluated by surgeons in comparison with the SpaceMouse, using two tasks in a virtual environment. The performance metrics show that surgeons using the prototype perform the task faster, with higher precision and fewer errors. This work forms the basis for an input device that can be used to control robotic devices and opens up the path toward more versatile robotic interventions.

## Appendix A: Statistical analysis

See Tables 1 and 2.

**Table 1:** *p*-value of the Wilcoxon test regarding the efficiency metrics, significant results are printed in bold (significance level = 1%)

Task	Time T	Path length PL	Working volume WV
Target	**7.4322e** $-$**09**	**1.6157e** $-$**12**	**3.453e** $-$**06**
TraceLine	0.93269	**9.7883e** $-$**11**	**1.7326e** $-$**08**

**Table 2:** *p*-value of the Wilcoxon test of the error metrics of task **Trace Line**, significant results are printed in bold (significance level = 1%)

Task	Error count	Error time	Error distance
TraceLine	**1.952e** $-$**09**	**4.794e** $-$**05**	**6.8079e** $-$**08**
